# Moving towards Routine Evaluation of Quality of Inpatient Pediatric Care in Kenya

**DOI:** 10.1371/journal.pone.0117048

**Published:** 2015-03-30

**Authors:** David Gathara, Rachael Nyamai, Fred Were, Wycliffe Mogoa, Jamlick Karumbi, Elesban Kihuba, Stephen Mwinga, Jalemba Aluvaala, Mercy Mulaku, Rose Kosgei, Jim Todd, Elizabeth Allen, Mike English, on behalf of the SIRCLE/Ministry of Health Hospital Survey Group

**Affiliations:** 1 KEMRI-Wellcome Trust Research Programme, Nairobi, Kenya; 2 Ministry of Health, Government of Kenya, Nairobi, Kenya; 3 Department of Pediatrics and Child Health, University of Nairobi, Nairobi, Kenya; 4 School of Pharmacy, University of Nairobi, Nairobi, Kenya; 5 Department of Obstetrics and Gynecology, University of Nairobi, Nairobi, Kenya; 6 Nuffield Department of Medicine & Department of Pediatrics, University of Oxford, Oxford, United Kingdom; 7 Department of Population Health, London School of Hygiene and Tropical Medicine, London, United Kingdom; 8 Department of Medical Statistics, London School of Hygiene and Tropical Medicine, London, United Kingdom; Nottingham University, UNITED KINGDOM

## Abstract

**Background:**

Regular assessment of quality of care allows monitoring of progress towards system goals and identifies gaps that need to be addressed to promote better outcomes. We report efforts to initiate routine assessments in a low-income country in partnership with government.

**Methods:**

A cross-sectional survey undertaken in 22 ‘internship training’ hospitals across Kenya that examined availability of essential resources and process of care based on review of 60 case-records per site focusing on the common childhood illnesses (pneumonia, malaria, diarrhea/dehydration, malnutrition and meningitis).

**Results:**

Availability of essential resources was 75% (45/61 items) or more in 8/22 hospitals. A total of 1298 (range 54–61) case records were reviewed. HIV testing remained suboptimal at 12% (95% CI 7–19). A routinely introduced structured pediatric admission record form improved documentation of core admission symptoms and signs (median score for signs 22/22 and 8/22 when form used and not used respectively). Correctness of penicillin and gentamicin dosing was above 85% but correctness of prescribed intravenous fluid or oral feed volumes for severe dehydration and malnutrition were 54% and 25% respectively. Introduction of Zinc for diarrhea has been relatively successful (66% cases) but use of artesunate for malaria remained rare. Exploratory analysis suggests considerable variability of the quality of care across hospitals.

**Conclusion:**

Quality of pediatric care in Kenya has improved but can improve further. The approach to monitoring described in this survey seems feasible and provides an opportunity for routine assessments across a large number of hospitals as part of national efforts to sustain improvement. Understanding variability across hospitals may help target improvement efforts.

## Introduction

Quality of care is assessed as one important output of health systems. Regular assessment allows monitoring of progress towards system goals and identifies gaps that need to be addressed to promote better health system outcomes [1,2]. Such monitoring however depends on an ability to measure quality, a multi-dimensional construct [3,4]. In high income settings, large routine patient level datasets are increasingly used to assess technical aspects of health service delivery, an example is the Clinical Research Practice Database (CRPD) in the United Kingdom. In low-resource settings data are very limited, often of poor quality [5–7], and rarely provide for individual patient level analyses. However, there is increasing recognition that data on both coverage and quality are essential to tracking progress of health systems [8]. Recognizing the need for better data and in line with their vision to provide quality health services to all, the Ministry of Health in Kenya initiated a process of large scale quality assessment of public hospital care through the Health Services, Implementation Research and Clinical Excellence (SIRCLE) Collaboration, a technical collaboration between the Ministry of Health, the University of Nairobi, and the KEMRI-Wellcome Trust Research Programme. This report examines the provision of pediatric inpatient services.

## Methods

### Context

In Kenya, the estimated under 5 mortality is 74/1000, with 31/1000 of these deaths occurring in the first 28 days after birth (i.e. the neonatal period) this is despite care for under-five’s being free in all public health facilities. In an effort to tackle this high mortality rate, the Kenyan government has produced and disseminated ‘*Basic Pediatric Protocols*’ consisting of clinical practice guidelines (CPGs)[9] since 2006, updating these in 2010. These guidelines are evidence-based, adapted from international and local disease specific guidelines, and focus on those illnesses responsible for more than 70% of pediatric admissions and deaths in public hospitals [7, 8]. Their introduction has been supported by an in-service training programme called “Emergency Triage Assessment and Treatment Plus admission care” (ETAT+, described in detail elsewhere) [10,11]. Training coverage of hospital clinical and nursing staff overall remains low (likely less than 15% workers) but approximately 60% of Kenyan medical undergraduates in the period 2008 to 2012 received a short form of this training[12]. Linked to the guidelines the government recommended in 2010 that hospitals use a structured pediatric admission record (PAR) demonstrated to improve documentation of core clinical characteristics at admission [13].

### Indicators

The resources required to deliver essential interventions to hospitalized children defined by government policies and the clinical guidelines provided the basic standards for subsequent quality assessment. Specific quality indicators were developed *a priori* and based on international[14] and local consensus of policy makers and professionals. Presence of resources (structure indicators) was evaluated across a set of six domains. Availability of each item was evaluated (score 0/1) and simple aggregate scores created for each domain ranging from 0 to the total of the items in the domain. A detailed description of the number and items in each of the domains is provided in **Table 1**. Further a cumulative summary score was computed as a total score of all items in the 6 domains (61 items).

**Table 1:** Structure items assessed for by domain.

**Equipment (n = 11)**	**Resources for supportive care (n = 11)**	**IV fluids and drugs (n = 12)**
Resuscitation couch	Oxygen	10% Dextrose
Bag-Valve-Mask device	Normal saline/ringers’ lactate	5% Dextrose
Resuscitation tray	Pediatric cannulae/scalp vein sets	Normal saline
Scales basin	IV giving sets, needles, syringes	Ringers lactate
Scales standing	Emergency room/area	ORS
Nebulizer/Spacer	Working Bag-Valve-Mask	Furosemide
Oxygen flow meter	Working suction equipment	Diazepam
Blood pressure Machine	NGT (gauges 8–10)	Phenobarbitone
Torch	Heat source	Adrenaline
Otoscope	Resuscitation equipment updated	Hydrocortisone
Chest tubes	Pediatric burette	Digoxin
		Nebulized or inhaled salbutamol
**Guidelines and wall charts (n = 9)**	**Vitamins, minerals and feeds (n = 8)**	**Availability of Antibiotics (n = 10)**
Basic Pediatric Protocol	Zinc tabs	Cotrimoxazole
Management of diarrhea	Vitamin A	Benzyl penicillin
Dosage guidelines	Vitamin K	Amoxicillin syrup
Management of pneumonia	Iron tabs/syrup	Gentamicin
Management of malaria	F100	(Flu)cloxacillin
PMTCT	F75	Ceftriaxone
Infant resuscitation	Term formula	Chloramphenicol
Newborn feeding	Pre-term formula	Ciprofloxacin
Newborn Resuscitation		Amoxicillin-clavulanate
		Ampicillin injection

NGT –Nasogastric tube; PMTCT-Prevention of Mother to Child Transmission; IV- Intra-venous

The total number of items assessed per domain form the total score of items expected from each domain

Items in the feeds and minerals, IV fluids and antibiotics were based on those that are listed in the essential medicines and commodities list.

Adoption of the structured pediatric admission record (PAR) was evaluated by determining the proportion of patients clerked on a PAR. A score representing the quality of medical documentation of the admission event was generated as the sum of scores (0/1) given for the documentation of specific symptoms (n = 11) and signs (n = 22) emphasized in guidelines. Median (inter-quartile range (IQR)) symptom and sign scores were then calculated for records from each hospital.

Process indicators for correct management of the common childhood illnesses were assessed for malaria, pneumonia, diarrhea/dehydration, malnutrition and meningitis. These indicators represent compliance with discrete steps within national guidelines[9] including: use of recommended disease severity categories (that determine management), use of recommended diagnostic tests, and correctness of prescriptions for treatment (drug and dosage, fluid or feed and administration rate). For the latter a 20% margin of error was allowed on the age and weight based recommendations provided in the guidelines. (**Table 2** describes the disease specific indicators in detail).

**Table 2:** Definition of the composite indicators of processes of care for each of the diseases.

Domain of care	Criteria for considering the composite indicator	Pneumonia	Dehydration	Malaria	Severe malnutrition	Meningitis
**Documentation**	All signs and symptoms required to make and appropriately classify disease severity	Cough, cyanosis, lower chest wall in-drawing respiratory rate, AVPU, ability to drink	Diarrhea, vomiting, capillary refill, sunken eyes and skin turgor, skin warm up to and AVPU	Fever, acidotic breathing, pallor, AVPU, unable to drink	Edema, visual assessment of degree of severe wasting	Convulsions, fever; level of consciousness
**Assessment**	Patient adequately assessed if all the following signs are assessed	Cough, cyanosis, lower chest wall in-drawing and respiratory rate	Sunken eyes and skin turgor (and duration of skin fold to return)	Level of consciousness, fever, acidotic breathing, pallor	Edema, visual assessment of degree of severe wasting	Convulsions, fever; level of consciousness (AVPU)
**Treatment**	Consistent with CPGs if the following key treatment was prescribed at the correct dose and frequency (and duration for rehydration therapy)	Crystalline penicillin 50,000units/kg/dose X 4 per day (+/−20%) and /or Gentamicin 7.5 mg/kg/day X 1 per day (+/−20%); correct treatment for disease severity	Severe dehydration cases prescribed fluids, Ringers lactate at 80–120 mls per kg or 56–120 mls per kg if bolus for shock management given; ORS prescribed for cases with some dehydration	Quinine loading dose prescribed; Quinine prescribed at 10mg/kg/dose (+/- 20%)	Feeds prescribed and of correct type; feed volume of 100–130 mls/kg/day (+/-20%) of F75	

AVPU- consciousness level documented as Alert, Verbal response, Pain response, Unresponsive; ORS-Oral rehydration solution

### Survey sites, data collection and sample size

The Ministry of Health identified 22 of 40 ‘internship training centres’, seeking geographical representation across Kenya, from a total population of Kenya’s 40 internship hospitals (see **S1 Fig.** for geographic location of hospitals). Internship hospitals provide supervised clinical practice to both graduate doctors and diploma level clinical officers[15] for one year prior to full registration. The Ministry of Health was interested in services in these centres as smaller hospitals are managed by these young clinicians on completion of their internships. Adopting the approach for cluster survey designs, and with 22 hospitals as the units of clustering, we estimated that retrieval of 60 case records per facility would provide samples for each common childhood illness (malaria, pneumonia and diarrhea/dehydration) in proportion to their admission fraction while contributing approximately 10 to 15 cases per diagnosis based on prior experience [13]. For disease specific indicators, and assuming a design effect of 1.5 based on previous work to account for clustering [13], reporting 50% or 10% correct performance with a precision of ±7.5% would be possible with a minimum of 12 and 4 cases respectively. The case records required were identified from ward registers by working backwards from 31^st^ May 2012 until the 60 cases closest to the survey were retrieved. Availability of resources was checked by observation against a standard checklist and compliance with process standards by careful examination of case records. Procedures are described in detail elsewhere [16].

### Analysis

For resources, we determined the proportion of hospitals in which a specific item was present to assess availability. Hospital and domain specific availability scores and the medians (and accompanying inter-quartile range and range) were calculated across hospitals. For case management indicators we report the proportion of all cases compliant with guidelines, this procedure providing a weighted estimate proportional to cases per hospital. The 95% confidence intervals (CI) were adjusted for clustering within hospitals.

We noted that performance for some process indicators varied greatly across hospitals. To demonstrate this, the median and range of hospital specific proportions for indicator compliance are presented and funnel plots are utilized to illustrate performance variation informed by 95% confidence intervals derived from our sample of 22 sites. In the latter case to constrain confidence intervals between the logical limits 0 and 1 for such indicators binomial exact methods were used.

All analyses were undertaken using Stata v11 (StataCorp, Texas, USA). Scientific and ethical approval for the study was obtained from the Kenya Medical Research Institute. The study involved review of routine case records and although these case records from which data were abstracted had patient names, data collected were anonymized and de-identified prior to analysis. This study was classified as an audit and therefore informed consent from the participants was not found necessary by the institutional ethics review committee. The Ministry of Health also approved the study and hospital management teams provided their assent prior to data collection.

## Results

### Resource availability

#### Pediatric staffing

All the internship hospitals surveyed had a dedicated ward/ward area for pediatric care with a median (IQR) patient-nurse ratio of 11 (7–22) and 26 (15–33) during the day and night respectively. Where workload data were available 11/15 (73%) hospitals were operating at more than 100% bed occupancy rate at the time of the survey. Sixteen hospitals had one pediatrician while 6 had two.

#### Organization of care

The median availability of essential equipment was 7 (IQR 6–8; max score = 11) with lowest availability items being a clinical torch, otoscope and chest drain tubes. Essential resources needed for supportive care were largely available, median availability score of 10 (IQR 8–12; max score = 12), however, resources for resuscitation were checked as up to date in 15/22 (68%) hospitals. Pediatric burettes for administering intravenous fluids accurately to infants and small children were available in only 8/22 (36%) of the hospitals. Guidelines and wall charts defining recommended management for common childhood illnesses had a median availability score of 3/9 (IQR 2–5; max score = 9) with newborn and infant resuscitation and feeding guidelines being available in less than 6/22 (27%) of the hospitals. Clinicians on duty on the days of survey had access to national pediatric protocol booklets in 16/22 (73%) of the hospitals.

**Fig 1:**
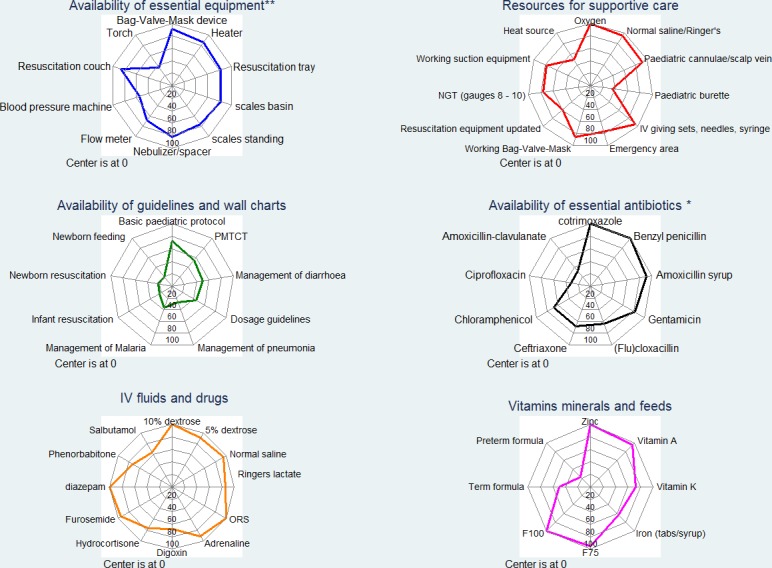
Organization of care and availability of essential resources. Percentage availability is determined as the proportion of 22 hospitals in which the specific item is present. 3 items available in less than 20% (4/22) of the hospitals were omitted. **Otoscope and torch omitted in essential equipment domain; * Ampicillin omitted in antibiotics domain.

**Fig 2:**
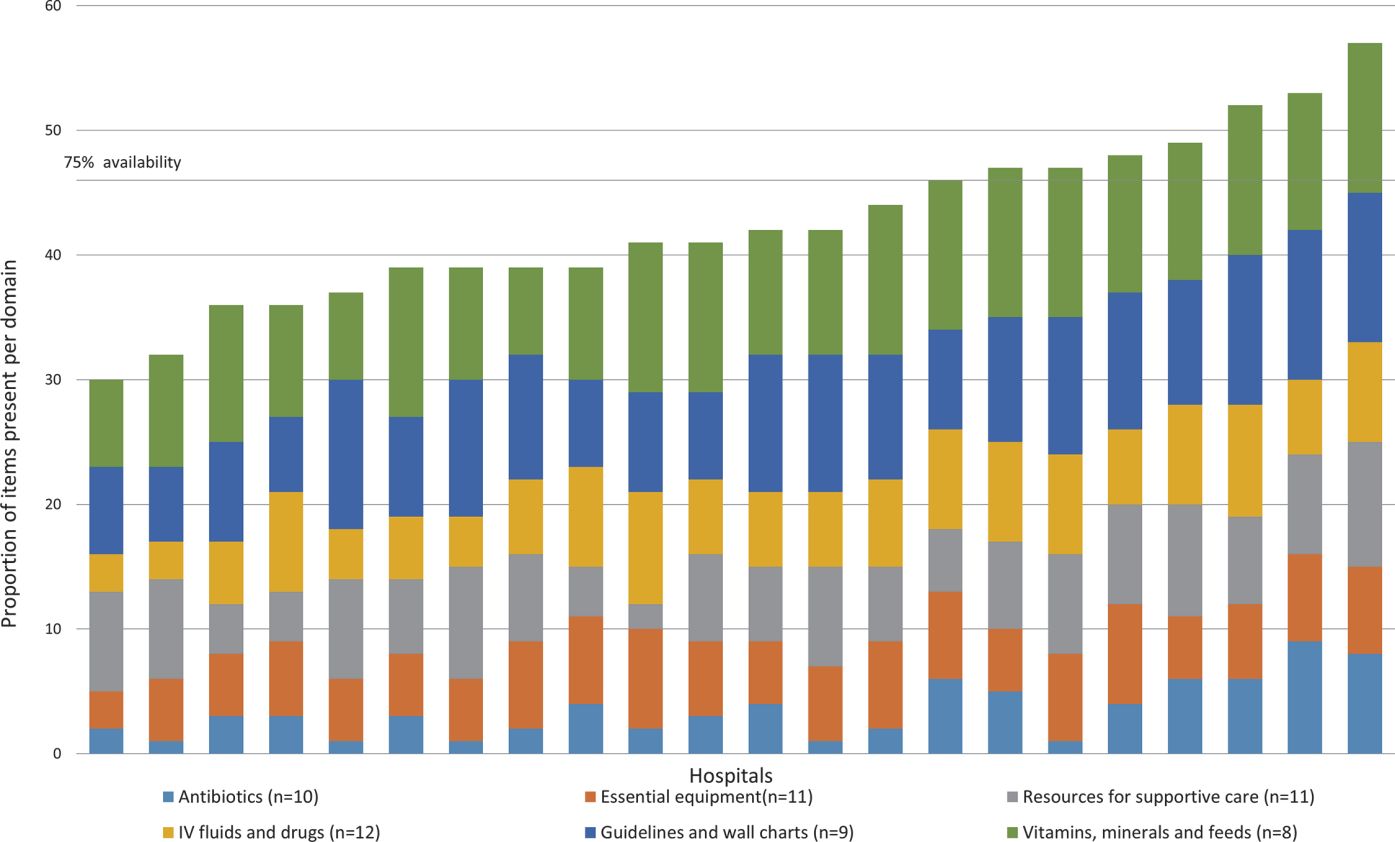
Cumulative availability of essential resources by domain and hospital. Proportion of items available per domain in each of the 6 domains (total is 100%) ordered across hospitals.

The median availability of essential antibiotics across all hospitals was 6 (IQR 5–8; max score = 10) with ampicillin injection, oral amoxicillin-clavulanic acid and oral ciprofloxacin (first line therapy for dysentery) available in less than 8/22 (36%) of the hospitals. Items in the IV fluids and drugs domain were available in more than 17/22 hospitals with a median availability of 11 (IQR 9–12; max score = 12) with the exception of digoxin and nebulized/inhaled salbutamol being available in 15/22 and 14/22 hospitals respectively. The median availability of vitamins, minerals and feeds was 6 (IQR 5–7; max score = 10) with term and pre-term formula being the least available in 11/22 and 5/22 hospitals respectively. Summarizing across all domains for the 61 essential resources, overall 8/22 hospitals had 75% (46/61) or more of these but availability ranged from a low of 49% (30/61) in one hospital to a maximum of 93% (57/61). Domain specific availability of structure items is presented in detail in **Fig. 1** while the overall availability is presented in **Fig. 2**. Detailed hospital specific results on resource availability are available in **S1 Table**.

### Process of care and case management

A total of 1298 case records were retrieved with a range of 54–61 records per hospital. Amongst these children 46% (597), 33% (433) and 21% (271) had diagnoses of pneumonia, malaria and diarrhea/dehydration respectively found in 1045 patients (details of the distribution of cases across hospitals are presented in **S2 Table**). A majority 747/1298 (58%) of the children were male while the median age was 14 (8–27) months. Although it is government policy that all children sick enough to be admitted to hospital should have a HIV test, this was only done in 156/1298 (12%, 95% CI 7–19) of the children.

#### Documentation

**Fig 3:**
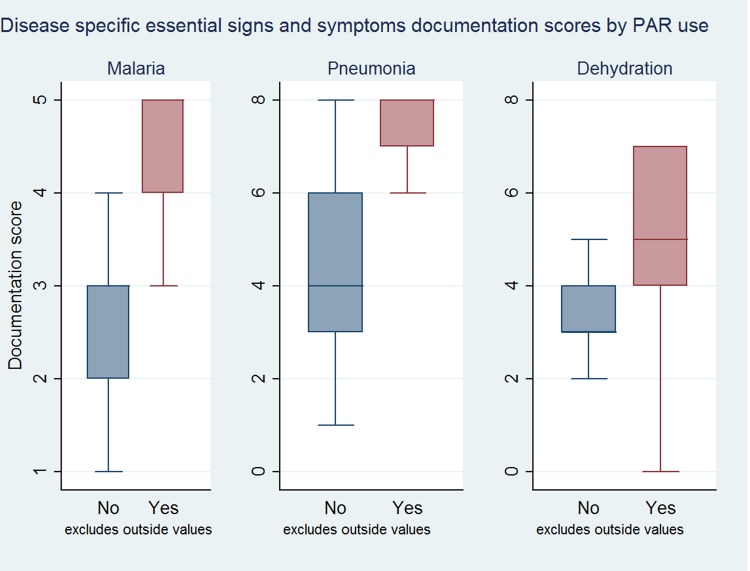
Documentation trends of disease specific key essential signs and symptoms. Documentation score of essential disease specific signs and symptoms stratified by PAR use for cases with no co-morbidities; x-axis is the documentation score with the disease total being the maximum value of x. *Outliers excluded.

The nationally recommended PAR was not used in 8/22 hospitals and usage varied from 13% to 100% in the remaining 14/22 hospitals (overall usage 588/1298, 43%, 95% CI 27%–61%). Pooling data across hospitals the median symptom (max = 11) and sign (max = 22) documentation scores were 6 vs 11 and 8 vs 20 when the PAR was and was not used by admitting clinicians respectively. This effect was still observed if analyses were restricted to diagnostic sub-groups (pneumonia, malaria and diarrhea/dehydration) **(Fig. 3**).

#### Disease specific process of care

**Fig 4:**
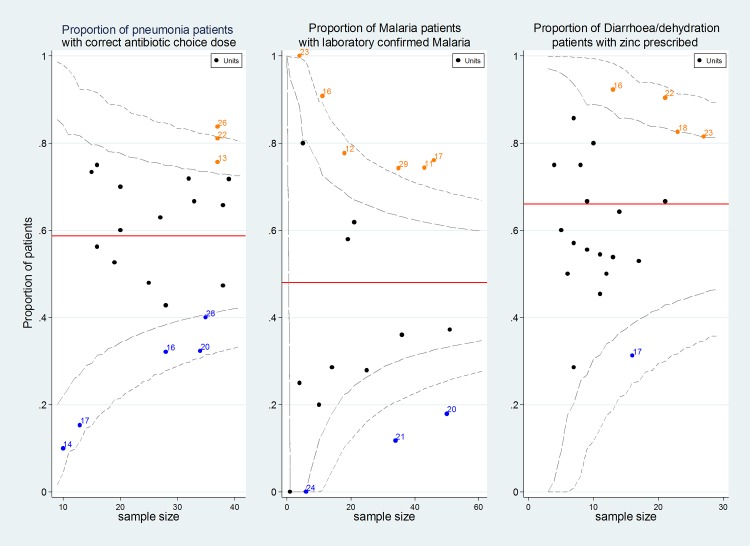
Variability of hospital performance across indicators. Variability funnel plots: X axis represents number of cases available for the indicator per hospital, Y axis represents the proportion of patients that achieve the indicator per hospital while the numbers against the data points are the hospital identifiers. The red line is the mean performance across hospitals while the dashed lines represent the 95% and 99% confidence intervals.

The use of guideline recommended severity classification was suboptimal for malaria and pneumonia at 44% and 73% respectively but high for dehydration at 92%. Further, correct treatment as recommended by guidelines (**Table 2**) varied greatly by disease ranging from 74% for malaria cases with quinine loading dose to 25% among malnutrition cases receiving correct type and volume of feeds prescribed. Of note was that only two cases were prescribed artemether and none had artesunate prescribed for malaria. In contrast to previous surveys, for diarrhoea/dehydration, overall zinc prescription was at 66%. Only a few children (n = 41/271) were prescribed metronidazole while no use of anti-emetics or anti-diarrheals was identified. Pooled process of care indicator performance by diagnosis is presented in **Table 3**(for hospital specific indicator performance see **S2 Table**). Substantial variation, likely not due to sampling error, was seen for HIV testing rates (range across hospitals 0 to 47% admissions, 95% CI for all hospital estimates 7% to 19%) and proportion of malaria cases with a laboratory confirmed diagnosis (**Fig. 4**)

**Table 3:** Performance of disease specific guideline recommended process indicators.

Disease	Indicator	n(%[95% CI])	Median (IQR) for hospital specific proportions
Malaria			
	Malaria cases	433	
	Use of guideline recommended severity classification	191 (44 [33–55])	100[86–100]
	Tested for malaria	396 (91 [87–95])	96[85–100]
	Lab confirmed malaria cases	208 (48 [33–63])	37[20–76]
	Malaria cases with Quinine loading dose	320 (74 [62–83])	72[30–86]
	Of those tested cases with slide positive results	208 (72 [57–83])	73[45–87]
	Prescribed quinine loading dose with negative result	48 (15 [8–26])	7.5[0–21]
	Malaria cases with weight and correct Quinine dose	243 (95 [92–97])	100[95–100]
Pneumonia			
	Pneumonia cases	597	
	Use of guideline recommended severity classification	433 (73 [63–80])	69[60–82]
	Gentamicin dose accuracy	232 (89 [82–93])	92[86–100]
	Correct antibiotic treatment as per severity	351 (59 [50–67])	62[43–72]
Dehydration			
	Dehydration cases	271	
	Use of guideline recommended severity classification	249 (92 [87–95])	100[86–100]
	zinc	179 (66 [57–74])	62[53–80]
	Dehydration cases with IV fluids	204 (75 [63–85])	82[69–100]
	Severe dehydration cases with IV fluids	90 (44 [33–56])	35[22–60]
	Correct fluid volume mls/kg for severe dehydration	54 (60 [43–75])	57[20–88]
	Cases with some dehydration given ORS	70 (56 [44–68])	50[33–80]
Malnutrition			
	Malnutrition cases	91	
	Severe wasting	56 (62 [36–82])	37[0–100]
	Edema of kwashiorkor	66 (73 [53–86])	71[33–100]
	Feeds prescribed and correct	51 (86 [69–95])	100[67–100]
	Correct feed type and volume mls/kg for all cases	25 (57 [39–73])	66[25–100]
Meningitis process indicators			
	Meningitis cases	115	
	Convulsions	96 (83 [71–91])	92[80–100]
	Fever	108 (94 [87–97])	100[100–100]
	AVPU	98 (85 [75–92])	100[78–100]
	Convulsions and fever	93 (81 [69–89])	92[75–100]
	LP ordered	62 (54 [39–68])	57[0–75]
	LP results available	21 (34 [21–49])	33[0–50]
HIV testing			
	HIV test done	156 (12 [7–19])	8[2–16]

*Indicators are defined in Table 1.

## Discussion

The primary purpose of the survey was to provide a national estimate of compliance with guidelines and identify gaps in the quality of care provided to children in hospital. Previous reports by our group and others have reported poor quality of care for common childhood illnesses in low income settings [5–7,17,18]. This assessment carried out in 22 hospitals is perhaps the first attempt to institute quality monitoring in partnership with government at reasonable scale and using well defined methods. While earlier work was less comprehensive there are indications that overall, the quality of pediatric care has improved compared to previous reports[19] [18]. For instance in the period 2002 and 2006, prescription of quinine loading dose, once daily gentamicin and appropriate prescription of fluids for severe dehydration was below 20% but above 60% in this survey[19]. In addition, there has been a dramatic fall in use of symptom relieving drugs not recommended in children such as anti-motility agents for diarrhea and considerable improvement in availability of appropriate feeds for severe malnutrition, use of correct fluid type for managing dehydration and documentation of illness in medical records linked to adoption of standardized pediatric admission records. These improvements are associated with the widespread dissemination of pediatric guidelines by the Ministry of Health in the form of Basic Pediatric Protocols together with more limited provision of ETAT+ [19] [9,10] and an increase in the number of pediatricians in public hospitals.

Despite these successes, essential resources were not uniformly available in hospitals providing supervised clinical practice to clinicians. There was limited access to some of the first line and second line antibiotics, and resources like pediatric burettes were available in less than 50% of the hospitals (no hospitals had infusion pumps). This may be of particular concern given recent debates over the safety of fluid administration in sick children in Africa [20]. Resource inadequacies together with absence of basic guidelines remain threats to provision of quality care. It is however encouraging that the ‘Basic Pediatric Protocols’ booklet that is provided to and held by clinicians individually was being used in over two-thirds of hospitals. One success of Kenya’s efforts to improve quality may be providing young clinicians with personal copies of these booklets during pre-service training. These seem to be valued by individuals in the early phase of their practice and offer an approach that may be more sustainable than providing multiple disease specific guidelines and wall charts.

A continued focus on improvement is required. For instance, continued effort is needed to ensure appropriate nutritional support to children admitted with complicated severe acute malnutrition[6] and determine childrens’ HIV status. It is encouraging to note adoption of some recent policy recommendations. Zinc was recommended as adjunctive therapy in 2010 to all children with diarrhea or vomiting and data suggest approximately 60% cases now receive it although use varies across place. In contrast, in 2010 WHO also recommended that Artesunate replace Quinine as first line therapy for malaria, but we did not find any use of Artesunate despite its adoption in local guidelines in 2011, the potential explanation being delays in national procurement of this drug.

Routine assessment of quality of care is increasingly recognized as an essential complement to, assessments of service coverage[8]. Our data provide insights on quality of pediatric care in Kenya using methods developed over a period of years that are based on a successful collaboration between researchers and government and that might support wider use at relatively low cost. As the use of similar protocol booklets linked to ETAT+ training is now occurring in Rwanda[21], and Uganda[22] in projects supported by The Royal College of Paediatrics and Child Health, with further use being discussed in Somaliland, Sierra Leone, and Zimbabwe, this approach to rapidly assessing quality of care might be used much more widely and allow countries to share experiences of what works. Ministries of health may also adopt some of the tools used in this work for evaluation of resource availability as is planned in Kenya as part of routine performance monitoring. This work has also prompted local efforts towards introduction of a minimum patient level dataset in the national health information system –District Health Information System (DHIS2) and adoption of quality of care indicators to inform the design of a pilot national Electronic Medical record.

In future, developing larger patient level datasets in a greater number of sites would allow for more comprehensive and representative quality of care assessment that appropriately identifies problems and prompts action in a timely manner within the health system. Ultimately working towards integrated electronic health record systems that are designed to capture data to populate quality indicators, combined with appropriate analyses, could support prompt feedback and supportive supervision to help drive quality improvement initiatives at scale and reduce the pronounced variability apparent at present. Researchers and the Ministry of Health are beginning to explore these possibilities in Kenya.

The data we report needs to be interpreted in light of the following limitations. Firstly, a relatively small number of hospitals were included and their selection by the Ministry of Health introduces a potential bias and we can only speculate about the state of the 18 other internship hospitals. However, a number of these 18 hospitals are in more remote parts of the country and anecdotal evidence would suggest these hospitals may be less well-resourced and staffed than those included in the report. Secondly, hospital specific results are based on relatively small sample sizes per hospital. Despite this, and the wide confidence intervals that result, funnel plots help illustrate the marked variability in quality of care observed across a relatively small number of hospitals. Thirdly, in work based on routine records we have to assume that all aspects of care that were delivered were documented, where documentation is poor, care may be interpreted as poor purely because of lack of data; a common generic limitation of such studies. Fourth, our choice of structure items in each domain may not be widely generalizable, however these items were selected to ensure consistency with recommendations in the ‘Basic Pediatric Protocols’ that draw on WHO’s essential medicines list which may be applicable to other low-resource limited settings with a similar epidemiological profile. Lastly, hospitals were aware of the survey although the records were retrieved from a period before the survey making our findings less prone to a Hawthorne-effect.

## Conclusion

Quality of pediatric care in Kenya has improved although care in some domains can be further improved. Without assessments such as the one conducted we remain ignorant of important health systems outputs and thus of whether investments in health are yielding the benefits we describe. Approaches for routine monitoring described in this survey provide an opportunity for performance monitoring and quality improvement across a large number of hospitals, as part of national efforts to improve health services. Such efforts would also enable exploration of variability across hospitals to be examined potentially helping to target improvement efforts.

## Supporting Information

S1 Fig Geographic location of hospitals. Red dots represent hospitals selected for the survey while the black lines represent county boundaries. Hospitals are clustered in the central and western regions consistent with where the majority of the Kenyan population lives.
(TIF)

S1 Table Hospital specific availability of essential resources. Availability of items per domain in each of the 6 domains across hospitals. 1 represents item available and 0 is item not available. The cumulative summary score is a total score of all items in the 6 domains (61 items).
(PDF)

S2 Table Hospital specific indicator performance. Proportion of children achieving an indicator within each hospital and overall pooled across hospital. Confidence intervals are adjusted for clustering.
(PDF)

## References

[1] Leatherman S, Ferris TG, Berwick D, Omaswa F, Crisp N (2010) The role of quality improvement in strengthening health systems in developing countries. International Journal of Quality in Health Care 22: 237–243. 10.1093/intqhc/mzq028 20543209

[2] Chan M, Kazatchkine M, Lob-Levyt J, Obaid T, Schweizer J, et al (2010) Meeting the demand for results and accountability: a call for action on health data from eight global health agencies. PLoS Medicine 7: e1000223 10.1371/journal.pmed.1000223 20126260 PMC2811154

[3] AbouZahr C, Boerma T (2005) Health information systems: the foundations of public health. Bull World Health Organization 83: 578–583. PMC2626318 16184276

[4] WHO (2008) Framework and Standards for Country Health Information Systems. Geneva, Switzerland: Health Metrics Network.

[5] English M, Esamai F, Wasunna A, Were F, Ogutu B, et al (2004) Assessment of inpatient paediatric care in first referral level hospitals in 13 districts in Kenya. Lancet 363: 1948–1953. 15194254 10.1016/S0140-6736(04)16408-8

[6] Gathara D, Opiyo N, Wagai J, Ntoburi S, Ayieko P, et al (2011) Quality of hospital care for sick newborns and severely malnourished children in Kenya: a two-year descriptive study in 8 hospitals. BMC Health Services Research 11: 307 10.1186/1472-6963-11-307 22078071 PMC3236590

[7] Reyburn H, Mwakasungula E, Chonya S, Mtei F, Bygbjerg I, et al (2008) Clinical assessment and treatment in paediatric wards in the north-east of the United Republic of Tanzania. Bull World Health Organization 86: 132–139. 18297168 10.2471/BLT.07.041723 PMC2647389

[8] Nesbitt RC, Lohela TJ, Manu A, Vesel L, Okyere E, et al (2013) Quality along the continuum: a health facility assessment of intrapartum and postnatal care in Ghana. PLoS One 8: e81089 10.1371/journal.pone.0081089 24312265 PMC3842335

[9] MoH (2012) Basic Pediatric Protocols Nairobi: Ministry of Health, Government of Kenya.

[10] Idoc-africa (2013) Emergency Triage Assessment and Treatment Plus admission care training.2013 [cited 2013 6th Novemeber]. Available: http://www.idoc-africa.org/. Accessed 2015 February 20.

[11] Irimu G, Wamae A, Wasunna A, Were F, Ntoburi S, et al (2008) Developing and introducing evidence based clinical practice guidelines for serious illness in Kenya. Archives of Disease in Childhood 93: 799–804. 10.1136/adc.2007.126508 18719161 PMC2654066

[12] English M, Wamae A, Nyamai R, Bevins B, Irimu G (2011) Implementing locally appropriate guidelines and training to improve care of serious illness in Kenyan hospitals: a story of scaling-up (and down and left and right). Archives of Disease in Childhood 96: 285–290. 10.1136/adc.2010.189126 21220265 PMC3039658

[13] Ayieko P, Ntoburi S, Wagai J, Opondo C, Opiyo N, et al (2011) A multifaceted intervention to implement guidelines and improve admission paediatric care in Kenyan district hospitals: a cluster randomised trial. PLoS Medicine 8: e1001018 10.1371/journal.pmed.1001018 21483712 PMC3071366

[14] Ntoburi S, Hutchings A, Sanderson C, Carpenter J, Weber M, et al (2010) Development of paediatric quality of inpatient care indicators for low-income countries—A Delphi study. BMC Pediatrics 10: 90 10.1186/1471-2431-10-90 21144065 PMC3022793

[15] Mbindyo P, Blaauw D, English M (2013) The role of Clinical Officers in the Kenyan health system: a question of perspective. Human Resources Health 11: 32 10.1186/1478-4491-11-32 23866692 PMC3724708

[16] Aluvaala J, Nyamai R, Were F, Wasunna A, Kosgei R, et al (2015) Assessment of neonatal care in clinical training facilities in Kenya. Archives of Disease in Childhood 100: 42–47. 10.1136/archdischild-2014-306423 25138104 PMC4283661

[17] Irimu GW, Gathara D, Zurovac D, Kihara H, Maina C, et al (2012) Performance of health workers in the management of seriously sick children at a Kenyan tertiary hospital: before and after a training intervention. PLoS One 7: e39964 10.1371/journal.pone.0039964 22859945 PMC3409218

[18] Mwinga S TM, Mweu E, English M. (2010) Report on the quality of paediatric and neonatal care in 17 government hospitals Nairobi: Ministry of Medical Services, Government of Kenya.

[19] English M, Gathara D, Mwinga S, Ayieko P, Opondo C, et al (2014) Adoption of recommended practices and basic technologies in a low-income setting. Archives of Disease in Childhood 99(5):452–6. 10.1136/archdischild-2013-305561 24482351 PMC3995214

[20] Maitland K, Kiguli S, Opoka RO, Engoru C, Olupot-Olupot P, et al (2011) Mortality after fluid bolus in African children with severe infection. New England Journal of Medicine 364: 2483–2495. 10.1056/NEJMoa1101549 21615299

[21] Tuyisenge L, Kyamanya P, Van Steirteghem S, Becker M, English M, et al (2014) Knowledge and skills retention following Emergency Triage, Assessment and Treatment plus Admission course for final year medical students in Rwanda: a longitudinal cohort study. Archives of Disease in Childhood 99(11):993–7. 10.1136/archdischild-2014-306078 24925893 PMC4198299

[22] RCPCH (2014) Improving the quality of hospital care for sick children in East Africa through ETAT+ training.2014 [cited 2014 1st August]. Available: http://www.rcpch.ac.uk/what-we-do/rcpch-international/volunteering-overseas/health-partnerships-scheme-grant-etat-east-africa. Accessed 2015 February 20.

