# Supplementary figures and images for: Moving towards Routine Evaluation of Quality of Inpatient Pediatric Care in Kenya
Source: PLoS One. 2015 Mar 30;10(3):e0117048. doi: 10.1371/journal.pone.0117048 (PMC4378956; doi:10.1371/journal.pone.0117048)

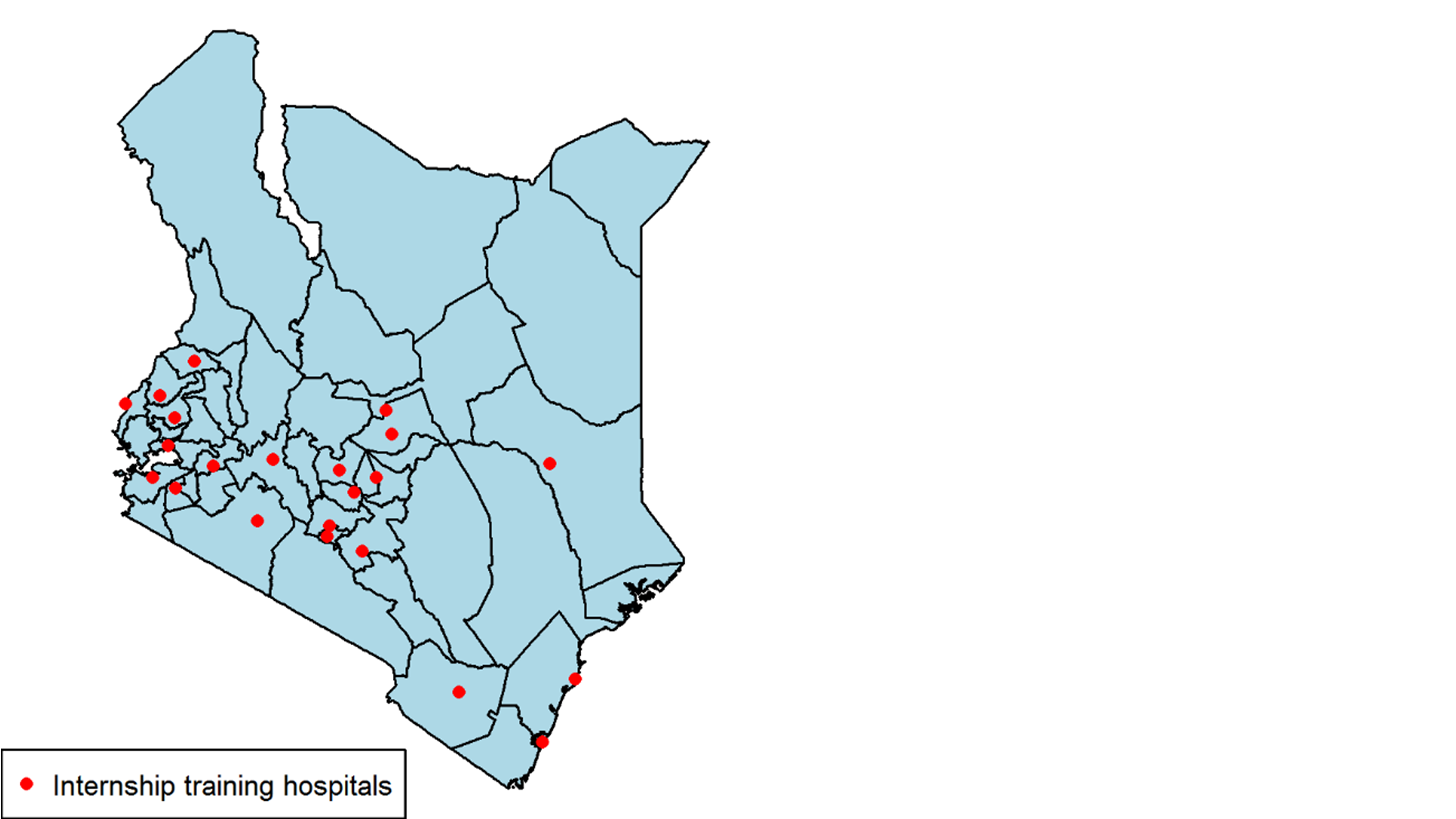

Supplement: S1 Fig — Red dots represent hospitals selected for the survey while the black lines represent county boundaries. Hospitals are clustered in the central and western regions consistent with where the majority of the Kenyan population lives. (TIF) [file pone.0117048.s001.tif]
