# Supplementary material for: Moving towards Routine Evaluation of Quality of Inpatient Pediatric Care in Kenya
Source: PLoS One. 2015 Mar 30;10(3):e0117048. doi: 10.1371/journal.pone.0117048 (PMC4378956; doi:10.1371/journal.pone.0117048)
Supplement: S2 Table — Proportion of children achieving an indicator within each hospital and overall pooled across hospital. Confidence intervals are adjusted for clustering. (PDF) [file pone.0117048.s003.pdf]

Hospital code	10	11	12	13	14	15	16	17	18	19	20	21	22	23	24	25	26	27	28	29	30	31	All hospitals pooled		
																							Cluster adjusted estimates n(%[95% CI])	Mediab (IQR) for hospital specific proportions	Range hospital specific estimates
Age in Months																									
Median (IQR)	18[11-27]	14[8-30]	11[6-30]	11[8-19]	14[7-24]	20[9-36]	16[7-29]	18[11-30]	16[9-30]	11[6-19]	20[12-34]	12[9-22]	14[7-22]	14[6-30]	9[5-18]	12[8-25]	16[8-28]	19[9-36]	9[6-18]	15[10-30]	17[10-37]	18[11-36]		14[8-27]	12_24
Weight																									
Weight documented	50 (83)	42 (71)	60 (100)	43 (72)	38 (67)	50 (93)	58 (97)	23 (39)	47 (77)	26 (44)	59 (98)	28 (56)	57 (95)	51 (85)	55 (92)	59 (98)	56 (93)	56 (93)	47 (78)	52 (87)	58 (98)	50 (83)	341 (79 [64 - 88])	89[67-100]	0_100
Gender																									
Male	35 (59)	39 (68)	39 (65)	35 (58)	29 (51)	30 (56)	34 (57)	34 (58)	38 (62)	32 (54)	32 (53)	27 (54)	36 (60)	34 (57)	35 (59)	35 (60)	34 (57)	32 (53)	36 (61)	34 (57)	34 (59)	33 (55)	747 (59 [57 - 60])	58[57-62]	53_68
PAR use																									
PAR used	0 (0)	0 (0)	60 (100)	8 (13)	0 (0)	0 (0)	57 (95)	30 (51)	22 (36)	38 (64)	25 (42)	0 (0)	44 (73)	10 (17)	0 (0)	54 (93)	57 (95)	55 (92)	2 (3)	1 (2)	53 (91)	42 (70)	558 (43 [27 - 61])	39[0-91]	0_100
length of stay																									
Median (IQR)	2[1-3]	2[1-3]	4[2-8]	3[2-5]	5[2-8]	2[2-5]	4[3-6]	3[2-4]	3[2-5]	3[2-5]	4[3-7]	3[2-4]	5[3-7]	4[3-7]	4[2-8]	4[2-10]	5[2-7]	4[2-6]	4[3-7]	3[2-6]	2[1-4]	3[2-4]		3[2-6]	3_8
History documentation (max 11)																									
Median (IQR)	4[3-7]	8[6-10]	11[11-11]	11[11-11]	11[11-11]	6[3-8]	11[11-11]	9[6-11]	8[5-11]	5[4-7]	7[5-11]	6[4-8]	10[4-11]	6[5-9]	4[2-5]	11[11-11]	4[2-4]	10[8-11]	4[3-7]	7[5-9]	11[11-11]	11[9-11]		9[5-11]	4_11
Examination documentation (max 22)																									
Median (IQR)	8[6-9]	7[6-9]	22[22-22]	19[18-21]	17[14-19]	8[6-9]	21[20-21]	12[7-16]	8[5-16]	11[4-15]	10[6-17]	6[5-8]	17[10-19]	8[6-12]	6[5-7]	21[21-22]	15[13-16]	12[9-13]	8[6-9]	8[6-9]	22[21-22]	19[7-21]		11[7-19]	6_22
Malaria																									
Malaria cases	51	43	18	4	21	36	11	46	25	1	50	34	0	4	6	5	0	14	10	35	0	19	433		
AVPU documented	34 (67)	37 (86)	18 (100)	4 (100)	18 (86)	31 (86)	11 (100)	38 (83)	13 (52)	1 (100)	36 (72)	13 (38)	0 (.)	3 (75)	4 (67)	5 (100)	0 (.)	13 (93)	8 (80)	30 (86)	0 (.)	13 (68)	330 (76 [66 - 84])	86[68-100]	38_100
Acidotic breathing documented	0 (0)	2 (5)	17 (94)	4 (100)	19 (90)	0 (0)	11 (100)	23 (50)	8 (32)	1 (100)	22 (44)	0 (0)	0 (.)	0 (0)	0 (0)	5 (100)	0 (.)	2 (14)	0 (0)	0 (0)	0 (.)	15 (79)	129 (30 [15 - 50])	32[0-94]	0_100
Pallor documented	50 (98)	41 (95)	18 (100)	3 (75)	21 (100)	35 (97)	11 (100)	45 (98)	25 (100)	1 (100)	47 (94)	30 (88)	0 (.)	4 (100)	6 (100)	5 (100)	0 (.)	14 (100)	7 (70)	34 (97)	0 (.)	17 (89)	414 (96 [93 - 97])	98[94-100]	70_100
Fever documented	48 (94)	43 (100)	18 (100)	4 (100)	21 (100)	35 (97)	11 (100)	46 (100)	23 (92)	1 (100)	48 (96)	31 (91)	0 (.)	4 (100)	2 (33)	5 (100)	0 (.)	14 (100)	8 (80)	33 (94)	0 (.)	18 (95)	413 (95 [92 - 97])	100[94-100]	33_100
Malaria process indicators																									
Tested for malaria	49 (96)	38 (88)	18 (100)	4 (100)	16 (76)	35 (97)	11 (100)	44 (96)	20 (80)	1 (100)	47 (94)	29 (85)	0 (.)	4 (100)	2 (33)	5 (100)	0 (.)	12 (86)	8 (80)	34 (97)	0 (.)	19 (100)	396 (91 [87 - 95])	96[85-100]	33_100
Lab confirmed malaria cases	19 (37)	32 (74)	14 (78)	1 (25)	13 (62)	13 (36)	10 (91)	35 (76)	7 (28)	0 (0)	9 (18)	4 (12)	0 (.)	4 (100)	0 (0)	4 (80)	0 (.)	4 (29)	2 (20)	26 (74)	0 (.)	11 (58)	208 (48 [33 - 63])	37[20-76]	0_100
Malaria cases with Quinine loading dose	44 (86)	41 (95)	13 (72)	1 (25)	16 (76)	30 (83)	11 (100)	42 (91)	14 (56)	0 (0)	39 (78)	20 (59)	0 (.)	4 (100)	1 (17)	1 (20)	0 (.)	9 (64)	3 (30)	15 (43)	0 (.)	16 (84)	320 (74 [62 - 83])	72[30-86]	0_100
Of the tested cases number with slide positive results	19 (66)	32 (91)	14 (82)	1 (33)	13 (87)	13 (45)	10 (91)	35 (95)	7 (58)	0 (0)	9 (35)	4 (50)	0 (.)	4 (100)	0 (.)	4 (80)	0 (.)	4 (80)	2 (33)	26 (87)	0 (.)	11 (65)	208 (72 [57 - 83])	73[45-87]	0_100
Of the tested cases number prescribed quinine loading dose with negative result	7 (16)	1 (2)	2 (15)	0 (0)	1 (6)	11 (37)	1 (9)	1 (2)	3 (21)	0 (.)	13 (33)	2 (10)	0 (.)	0 (0)	0 (0)	0 (0)	0 (.)	0 (0)	1 (33)	0 (0)	0 (.)	5 (31)	48 (15 [8 - 26])	7.5[0 - 21]	0_37
Malaria cases with correct Quinine dose mg/kg	35 (95)	28 (97)	12 (100)	0 (.)	10 (83)	24 (89)	7 (100)	17 (94)	12 (100)	0 (.)	40 (98)	11 (85)	0 (.)	4 (100)	1 (100)	1 (100)	0 (.)	9 (100)	2 (100)	16 (100)	0 (.)	14 (100)	243 (95 [92 - 97])	100[95-100]	83_100
Pneumonia process indicators																									
Pneumonia cases	15	16	32	37	10	19	28	13	20	27	34	28	37	20	38	39	37	38	35	16	33	25	597		
Cough documented	14 (93)	16 (100)	32 (100)	37 (100)	10 (100)	18 (95)	28 (100)	11 (85)	19 (95)	22 (81)	33 (97)	27 (96)	31 (84)	19 (95)	32 (84)	39 (100)	29 (78)	38 (100)	31 (89)	16 (100)	33 (100)	25 (100)	560 (94 [89 - 97])	97[89-100]	78_100
Respiratory rate documented	13 (87)	10 (63)	32 (100)	36 (97)	7 (70)	14 (74)	23 (82)	3 (23)	10 (50)	6 (22)	17 (50)	20 (71)	20 (54)	14 (70)	3 (8)	34 (87)	21 (57)	35 (92)	13 (37)	14 (88)	33 (100)	13 (52)	391 (65 [51 - 78])	70[50-87]	8_100
Lower chest wall in-drawing documented	14 (93)	3 (19)	32 (100)	37 (100)	10 (100)	7 (37)	28 (100)	10 (77)	16 (80)	16 (59)	29 (85)	15 (54)	32 (86)	10 (50)	23 (61)	39 (100)	35 (95)	31 (82)	28 (80)	11 (69)	33 (100)	22 (88)	481 (81 [70 - 88])	84[61-100]	19_100
Central cyanosis documented	12 (80)	14 (88)	32 (100)	36 (97)	10 (100)	9 (47)	28 (100)	12 (92)	13 (65)	21 (78)	25 (74)	21 (75)	31 (84)	16 (80)	31 (82)	39 (100)	35 (95)	37 (97)	34 (97)	14 (88)	33 (100)	22 (88)	525 (88 [81 - 93])	88[80-97]	47_100
AVPU documented	10 (67)	15 (94)	32 (100)	37 (100)	8 (80)	14 (74)	28 (100)	11 (85)	9 (45)	18 (67)	27 (79)	8 (29)	32 (86)	14 (70)	17 (45)	39 (100)	34 (92)	35 (92)	30 (86)	15 (94)	33 (100)	16 (64)	482 (81 [69 - 89])	86[67-94]	29_100
Grunting documented	9 (60)	1 (6)	32 (100)	37 (100)	10 (100)	2 (11)	28 (100)	8 (62)	12 (60)	12 (44)	17 (50)	4 (14)	26 (70)	5 (25)	2 (5)	39 (100)	35 (95)	12 (32)	17 (49)	4 (25)	33 (100)	21 (84)	366 (61 [44 - 76])	60[25-100]	5_100
Ability to drink documented	2 (13)	0 (0)	32 (100)	37 (100)	9 (90)	0 (0)	28 (100)	8 (62)	5 (25)	17 (63)	13 (38)	8 (29)	30 (81)	3 (15)	5 (13)	39 (100)	31 (84)	13 (34)	5 (14)	1 (6)	33 (100)	20 (80)	339 (57 [39 - 73])	50[14-90]	0_100
Crystalline penicillin dose accurracy	10 (100)	10 (83)	23 (85)	23 (100)	6 (100)	12 (92)	22 (100)	1 (100)	13 (100)	10 (91)	28 (88)	6 (100)	31 (97)	14 (93)	24 (77)	30 (91)	28 (90)	29 (94)	22 (96)	12 (100)	21 (100)	20 (87)	395 (92 [88 - 95])	95[90-100]	77_100
Gentamicin dose accuracy	4 (100)	8 (80)	18 (72)	8 (100)	5 (100)	7 (88)	17 (89)	0 (.)	12 (100)	5 (83)	22 (100)	4 (67)	13 (87)	12 (86)	13 (93)	18 (100)	14 (74)	25 (89)	4 (100)	7 (100)	4 (100)	12 (92)	232 (89 [82 - 93])	92[86-100]	67_100
Correct antibiotic treatment as per severity classfication	11 (73)	9 (56)	23 (72)	28 (76)	1 (10)	10 (53)	9 (32)	2 (15)	14 (70)	17 (63)	11 (32)	12 (43)	30 (81)	12 (60)	25 (66)	28 (72)	31 (84)	18 (47)	14 (40)	12 (75)	22 (67)	12 (48)	351 (59 [50 - 67])	62[43-72]	10_84
Dehydration																									
Dehydration cases	4	6	9	8	12	9	13	16	23	21	11	7	21	27	7	5	13	11	17	10	7	14	271		
Sunken eyes documented	2 (50)	0 (0)	9 (100)	8 (100)	12 (100)	3 (33)	13 (100)	3 (19)	6 (26)	2 (10)	9 (82)	2 (29)	0 (0)	12 (44)	3 (43)	5 (100)	0 (0)	3 (27)	3 (18)	3 (30)	7 (100)	7 (50)	112 (41 [27 - 58])	38[19-100]	0_100
Skin pinch documented	2 (50)	0 (0)	7 (78)	7 (88)	6 (50)	4 (44)	13 (100)	9 (56)	10 (43)	4 (19)	5 (45)	0 (0)	14 (67)	10 (37)	1 (14)	4 (80)	4 (31)	5 (45)	7 (41)	3 (30)	7 (100)	7 (50)	129 (48 [37 - 58])	45[31-67]	0_100
zinc prescribed	3 (75)	3 (50)	5 (56)	6 (75)	6 (50)	6 (67)	12 (92)	5 (31)	19 (83)	14 (67)	6 (55)	2 (29)	19 (90)	22 (81)	6 (86)	3 (60)	7 (54)	5 (45)	9 (53)	8 (80)	4 (57)	9 (64)	179 (66 [57 - 74])	62[53-80]	29_92
Dehydration cases with IV fluids prescribed	3 (75)	5 (83)	3 (33)	7 (88)	11 (92)	7 (78)	13 (100)	11 (69)	19 (83)	12 (57)	9 (82)	7 (100)	16 (76)	26 (96)	7 (100)	5 (100)	3 (23)	9 (82)	6 (35)	10 (100)	7 (100)	8 (57)	204 (75 [63 - 85])	82[69-100]	23_100
Severe dehydration cases with IV fluids	2 (67)	1 (20)	1 (33)	0 (0)	6 (55)	2 (29)	8 (62)	4 (36)	6 (32)	4 (33)	4 (44)	1 (14)	15 (94)	14 (54)	5 (71)	3 (60)	0 (0)	2 (22)	3 (50)	3 (30)	1 (14)	5 (63)	90 (44 [33 - 56])	35[22-60]	0_94
Severe dehydration cases with correct fluid volume mls/kg	1 (50)	0 (0)	1 (100)	0 (.)	3 (50)	1 (50)	7 (88)	1 (25)	5 (83)	0 (0)	3 (75)	0 (0)	13 (87)	9 (64)	1 (20)	2 (67)	0 (.)	2 (100)	0 (0)	3 (100)	1 (100)	1 (20)	54 (60 [43 - 75])	57[20-88]	0_100
Cases with some dehydration given ORS	1 (100)	3 (75)	3 (50)	5 (63)	4 (67)	2 (33)	0 (0)	2 (33)	7 (50)	4 (44)	6 (100)	4 (80)	5 (100)	7 (88)	0 (0)	0 (0)	2 (25)	3 (43)	1 (33)	2 (50)	6 (100)	3 (60)	70 (56 [44 - 68])	50[33-80]	0_100
Malnutrition																									
Malnutrition cases	2	1	7	8	10	5	3	0	4	2	6	1	1	5	2	15	1	3	3	9	0	3	91		
Severe wasting documented	0 (0)	1 (100)	7 (100)	8 (100)	10 (100)	2 (40)	3 (100)	0 (.)	3 (75)	0 (0)	2 (33)	0 (0)	0 (0)	3 (60)	0 (0)	15 (100)	0 (0)	0 (0)	0 (0)	0 (0)	0 (.)	2 (67)	56 (62 [36 - 82])	37[0-100]	0_100
Oedema of kwashiokor documented	1 (50)	1 (100)	7 (100)	8 (100)	6 (60)	4 (80)	3 (100)	0 (.)	3 (75)	0 (0)	3 (50)	0 (0)	1 (100)	5 (100)	0 (0)	15 (100)	0 (0)	3 (100)	1 (33)	3 (33)	0 (.)	2 (67)	66 (73 [53 - 86])	71[33-100]	0_100
Cases with feeds prescribed and of correct type	1 (100)	1 (100)	1 (50)	4 (100)	1 (25)	4 (100)	0 (0)	0 (.)	2 (67)	0 (.)	6 (100)	0 (.)	1 (100)	2 (67)	1 (100)	14 (93)	1 (100)	3 (100)	1 (100)	6 (100)	0 (.)	2 (100)	51 (86 [69 - 95])	100[67-100]	0_100
Cases with orrect feed type and volume mls/kg	1 (100)	0 (.)	0 (0)	3 (75)	0 (0)	4 (100)	0 (.)	0 (.)	1 (50)	0 (.)	2 (33)	0 (.)	0 (.)	2 (100)	0 (.)	7 (58)	1 (100)	0 (0)	1 (100)	1 (25)	0 (.)	2 (100)	25 (57 [39 - 73])	66[25-100]	0_100
Meningitis process indicators																									
Meningitis cases	5	1	5	5	3	12	5	6	8	3	9	1	2	1	6	7	11	13	4	7	1	0	115		
Convulsions documented	4 (80)	1 (100)	5 (100)	5 (100)	3 (100)	11 (92)	5 (100)	5 (83)	7 (88)	1 (33)	9 (100)	0 (0)	2 (100)	1 (100)	2 (33)	7 (100)	8 (73)	12 (92)	1 (25)	6 (86)	1 (100)	0 (.)	96 (83 [71 - 91])	92[80-100]	0_100
Fever documented	5 (100)	1 (100)	5 (100)	5 (100)	3 (100)	12 (100)	5 (100)	6 (100)	7 (88)	3 (100)	9 (100)	1 (100)	2 (100)	1 (100)	4 (67)	7 (100)	9 (82)	12 (92)	3 (75)	7 (100)	1 (100)	0 (.)	108 (94 [87 - 97])	100[100-100]	67_100
AVPU documented	5 (100)	1 (100)	5 (100)	5 (100)	3 (100)	12 (100)	5 (100)	4 (67)	6 (75)	3 (100)	7 (78)	0 (0)	2 (100)	1 (100)	2 (33)	7 (100)	9 (82)	11 (85)	4 (100)	5 (71)	1 (100)	0 (.)	98 (85 [75 - 92])	100[78-100]	0_100
Convulsions and fever documented	4 (80)	1 (100)	5 (100)	5 (100)	3 (100)	11 (92)	5 (100)	5 (83)	6 (75)	1 (33)	9 (100)	0 (0)	2 (100)	1 (100)	2 (33)	7 (100)	7 (64)	11 (85)	1 (25)	6 (86)	1 (100)	0 (.)	93 (81 [69 - 89])	92[75-100]	0_100
LP ordered	4 (80)	0 (0)	0 (0)	3 (60)	0 (0)	12 (100)	3 (60)	3 (50)	6 (75)	0 (0)	6 (67)	1 (100)	2 (100)	1 (100)	3 (50)	4 (57)	3 (27)	6 (46)	0 (0)	5 (71)	0 (0)	0 (.)	62 (54 [39 - 68])	57[0-75]	0_100
LP results available	2 (50)	0 (.)	0 (.)	1 (33)	0 (.)	6 (50)	1 (33)	1 (33)	4 (67)	0 (.)	1 (17)	0 (0)	2 (100)	0 (0)	0 (0)	1 (25)	1 (33)	1 (17)	0 (.)	0 (0)	0 (0)	0 (.)	21 (34 [21 - 49])	33[0-50]	0_100
HIV testing																									
HIV test done	0 (0)	0 (0)	28 (47)	6 (10)	1 (2)	3 (6)	16 (27)	0 (0)	10 (16)	0 (0)	25 (42)	0 (0)	5 (8)	13 (22)	6 (10)	7 (12)	1 (2)	17 (28)	3 (5)	9 (15)	1 (2)	5 (8)	156 (12 [7 - 19])	8[2-16]	0_47
